# Use of Patient-Derived Organoids as a Treatment Selection Model for Colorectal Cancer: A Narrative Review

**DOI:** 10.3390/cancers14041069

**Published:** 2022-02-20

**Authors:** Sara Furbo, Paulo César Martins Urbano, Hans Henrik Raskov, Jesper Thorvald Troelsen, Anne-Marie Kanstrup Fiehn, Ismail Gögenur

**Affiliations:** 1 Center for Surgical Science, Department of Surgery, Zealand University Hospital, Lykkebækvej 1, 4600 Køge, Denmark; sarafp@regionsjaelland.dk (S.F.); pamar@regionsjaelland.dk (P.C.M.U.); hansr@regionsjaelland.dk (H.H.R.); ankf@regionsjaelland.dk (A.-M.K.F.); 2 Department of Science and Environment, Roskilde University, Universitetsvej 1, 4000 Roskilde, Denmark; troelsen@ruc.dk; 3 Enhanced Perioperative Oncology (EPeOnc) Consortium, Zealand University Hospital, 4600 Køge, Denmark; 4 Department of Pathology, Zealand University Hospital, Sygehusvej 10, 4000 Roskilde, Denmark; 5 Department of Clinical Medicine, University of Copenhagen, Blegdamsvej 3B, 2200 København, Denmark

**Keywords:** colorectal cancer, patient-derived organoids, personalized treatment, treatment selection model, tumor-infiltrating lymphocytes

## Abstract

**Simple Summary:**

Colorectal cancer (CRC) is the third most common type of cancer globally. Despite successful treatment, it has a 40% chance of recurrence within five years after surgery. While neoadjuvant chemotherapy is offered for stage IV cancers, it comes with a risk of resistance and disease progression. CRC tumors vary biologically, recur frequently, and pose a significant risk for cancer-related mortality, making it increasingly relevant to develop methods to study personalized treatment. A tumor organoid is a miniature, multicellular, and 3D replica of a tumor in vitro that retains its characteristics. Here, we discuss the current methods of culturing organoids and the correlation of drug response in organoids with clinical responses in patients. This helps us to determine whether organoids can be used for treatment selection in a clinical setting. Based on the studies included, there was a strong correlation between treatment responses of organoids and clinical treatment responses.

**Abstract:**

Surgical resection is the mainstay in intended curative treatment of colorectal cancer (CRC) and may be accompanied by adjuvant chemotherapy. However, 40% of the patients experience recurrence within five years of treatment, highlighting the importance of improved, personalized treatment options. Monolayer cell cultures and murine models, which are generally used to study the biology of CRC, are associated with certain drawbacks; hence, the use of organoids has been emerging. Organoids obtained from tumors display similar genotypic and phenotypic characteristics, making them ideal for investigating individualized treatment strategies and for integration as a core platform to be used in prediction models. Here, we review studies correlating the clinical response in patients with CRC with the therapeutic response in patient-derived organoids (PDO), as well as the limitations and potentials of this model. The studies outlined in this review reported strong associations between treatment responses in the PDO model and clinical treatment responses. However, as PDOs lack the tumor microenvironment, they do not genuinely account for certain crucial characteristics that influence therapeutic response. To this end, we reviewed studies investigating PDOs co-cultured with tumor-infiltrating lymphocytes. This model is a promising method allowing evaluation of patient-specific tumors and selection of personalized therapies. Standardized methodologies must be implemented to reach a “gold standard” for validating the use of this model in larger cohorts of patients. The introduction of this approach to a clinical scenario directing neoadjuvant treatment and in other curative and palliative treatment strategies holds incredible potential for improving personalized treatment and its outcomes.

## 1. Introduction

Colorectal cancer (CRC) is the third most common cancer worldwide and is the second leading cause of cancer-related deaths [1]. The first line of treatment against stage I–III CRC is surgical resection combined with adjuvant chemotherapy, which is often 5-fluorouracil (5-FU) and oxaliplatin [2,3,4]. Minimally invasive surgical procedures have reduced morbidity and the length of hospital stays and have improved quality of life compared to that observed with open surgery [5,6]. Even if a patient undergoes a successful curative-intended treatment for CRC, there is an up to 40% risk of recurrence within five years due to residual disease [2,3]. 

Curative surgery is rarely an option for patients with stage IV cancer. However, some patients receive neoadjuvant chemotherapy and/or radiation therapy to downstage the tumor, with the aim of subsequent resection and reduction of local and systemic recurrence [3,7]. The recent introduction of immune checkpoint inhibitors has also shown promising results in certain subgroups of patients with CRC [8,9].

Although cytotoxic chemotherapy is rarely curative, it may improve the progression-free survival rates and the efficacy of immunotherapy. Conversely, chemotherapy may also lead to resistance and disease progression caused by the influence of the tumor microenvironment (TME) and especially that of CRC stem cells, which are responsible for inter-tumoral heterogeneity and clonal diversity [2,10,11,12]. Due to the diverse biology of CRC tumors, high recurrence rates, and risk of cancer-related deaths, there is an ever-increasing need for improved personalized treatment strategies to reduce the morbidity and mortality of CRC.

Currently, monolayer cultures are the preferred method for drug screening in cancer research. However, this method entails several limitations, including the lack of cancer-cell heterogeneity and the absence of TME, both affecting drug responses and not truly displaying clinically observed responses [13,14,15,16,17]. To examine personalized tumors and drug response in vivo—including the sensitivity and resistance to chemotherapy and chemotherapeutic combinations—carcinogen-induced or genetically engineered murine models are used most often [14,18,19,20,21,22,23]. These experiments are conducted in immune-deficient murine models or patient-derived xenografts, which are associated with major financial, ethical, laborious, and logistic challenges [24,25,26,27]. In view of overcoming the aforementioned limitations, the use of organoids is increasing. 

Organoids are miniature, multicellular, and three-dimensional (3D) models of an organ or tumor produced in vitro that are established from pluripotent stem cells or progenitor cells from an organ. Organoids are capable of self-renewal and organize themselves to resemble the original organ or tumor in both structure and function [28]. Organoids derived from human tumors retain the tumor’s heterogeneity and mutational features and imitate the realistic microanatomy. These organoids are defined as patient-derived organoids (PDO) [29,30]. Several studies have used PDOs from gastric, ovarian, liver, and breast cancers as drug screening models [24,31,32,33,34]. The use of PDOs is more cost beneficial, less time consuming, and allows high-throughput screening compared to mouse models; nonetheless, it is important to note that they are devoid of the TME [13,14]. Recent studies have investigated the possibility of co-culturing PDOs with mesenchymal and immune cells such as tumor-infiltrated lymphocytes (TILs), enabling the simulation of TME [35,36].

In this review, we present the current methods of culturing organoids and the research correlating clinical patient response with drug response in PDOs and co-culture PDOs obtained from CRC. In our attempt to investigate their potential as treatment selection and treatment prediction models in the future, we also evaluate the limitations and potentials of using these methods in a clinical setting.

## 2. Reviewing Methods

The following section reviews the methods used to establish PDOs and PDOs co-cultured with TILs. In addition, the setup of drug screening assays is described.

### 2.1. Establishment of PDOs

**Figure 1:**
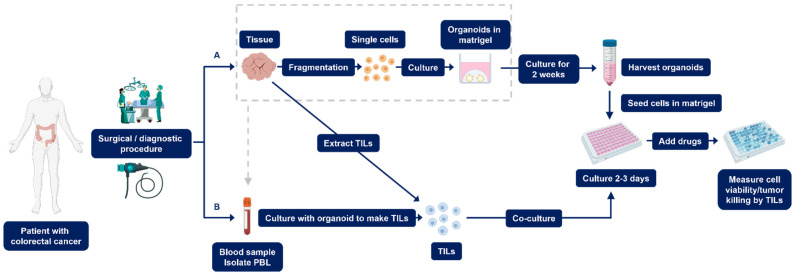
A flowchart depicting the drug screening methods using PDOs (**A**) and co-culture PDOs (**B**). (**A**) The tissue is obtained by either surgical or diagnostic procedures and fragmented to obtain single cells. These cells are cultured in an extracellular matrix for two weeks. Subsequently, the organoids are harvested and seeded in tissue culture plates and allowed to recover for 2–3 days. Next, drugs are added to the organoids and cell viability is measured. (**B**) The TILs are either obtained from PBLs after co-culturing with organoids or extracted from the tissue. They are then co-cultured with the organoids and subsequently exposed to the drugs. Organoid-killing by TILs is measured. PBL, peripheral blood lymphocytes; TILs, tumor-infiltrating lymphocytes; PDOs, patient-derived organoids.

Studies have shown that PDOs can be generated from primary CRC (pCRC) and metastatic CRC (mCRC) in 90% and 71% of cases, respectively [37,38]. To generate a PDO from a biopsy, the tissue is washed with phosphate-buffered saline and cut into smaller pieces [35,39]. Next, the tissue is enzymatically digested to generate even smaller fragments using enzymes such as collagenase and dispase. Fragments are eventually dissociated with TrypLE Express and DNase I into single cells, which are subsequently transferred to a gel-based extracellular matrix (Figure 1A) [35,39,40]. This gel matrix is composed of extracellular matrix proteins, essentially collagen and laminins, which support cell proliferation and its organization into 3D cultures [41].

An organoid-specific medium with suitable growth factors is used to culture organoids and to allow cell growth (Table S1) [14,35,42]. Supplementation of WNT is essential for the establishment of organoids obtained from healthy tissue. A reason for this is that 90% of the CRC tumors have a mutation in the APC gene, resulting in constitutive signaling through the WNT pathway, which is not the case for healthy tissue [35,43].

Once established, it is possible to freeze and store the organoid’s lines in research biobanks. Moreover, it is even possible to fragment the tissue, freeze the fragments, and generate organoids later. In several studies, even after a freeze and thaw cycle, organoids were found to retain their original characteristics on the gene expression level. However, this might lead to cell death [29,43,44]. Nevertheless, this underscores the convenience of using PDOs as a tool in experimental cancer research, drug development, and clinical research, among others [29,45].

#### 2.1.1. Quality Control to Ensure Compliance

Ideally, the mutational profile and protein expression patterns of PDOs should be investigated at each passage and before initiating a drug-screening experiment to ensure that the model maintains the characteristics of the original tumor. Histological assessment of whether the PDOs match their corresponding tumor should be performed in the initial stages of their establishment. This should ideally be performed blinded by a pathologist evaluating hematoxylin and eosin (H&E) stains of both the tumor tissue from the original diagnostic biopsies or surgical specimens and the organoids [46]. 

**Figure 2:**
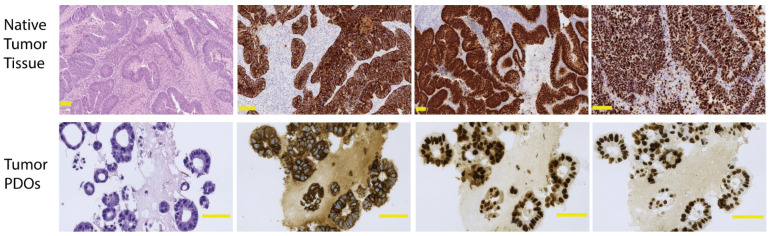
Immunohistochemical (IHC) analysis of patient-derived organoids and its matching sigmoid colon cancer tissue. H&E is the classical staining used to verify the morphological similarity, pan-CK is a broad-spectrum epithelial marker, CDX2 is expressed in the majority of colorectal adenocarcinomas, although not restricted to this organ, and Ki67 is a cell proliferation marker. The scanned slides are gamma-adjusted to obtain better discrimination and all bars represent 100 μm. Images are obtained from a resected tumor of a patient operated on at Zealand University Hospital. The patient provided verbal and written consent for the use of the images. H&E, hematoxylin and eosin; pan-CK, pan-cytokeratin; CDX2, caudal type homeobox 2.

Moreover, the expression patterns of proteins can be correlated across parent tissue and organoids by immunohistochemistry (IHC) analysis. The IHC analysis utilizing various markers such as pan-cytokeratin, caudal type homeobox 2 (CDX2), cytokeratin 20 (CK20), and Ki67 has been previously described [46,47,48,49]. Pan-cytokeratin differentiates epithelial cells from non-epithelial cells, while CDX2 and CK20 are more specific and react with the colorectal epithelium, although expression is not restricted to this organ. Ki67 is a marker of proliferating cells [50]. An example of H&E and IHC stained sections of a tissue section from sigmoid colon cancer and its generated organoids is shown in Figure 2. Both the original tumor and the organoids consist of glandular structures lined by the intestinal epithelial cells. IHC staining shows similar reactions towards pan-cytokeratin and CDX2, and the proliferation index is within the same range. This emphasizes that, to a large extent, PDOs can be considered as a realistic imitation of a tumor. 

Sequencing is used to determine the mutations and copy number alterations/variations (CNA/CNV) of the genome or exome. Comparison of mutations and CNAs across the blood, tumor tissue, and PDOs of the same patient are especially crucial to ensure that the mutations and CNA/CNVs are retained in the organoid as well as over multiple organoid passages. Performing this step enables one to discard the organoids if they do not recapitulate the mutations and CNAs observed in the corresponding parent tissue [46,48]. It is already known that, compared to corresponding tumors, up to 96% of the mutational profiles are displayed in the PDOs [47,49], thereby maintaining the genetic diversity of tumors [37]. 

Some investigators perform single nucleotide polymorphisms (SNP) analysis on the DNA obtained from organoids and blood, thereby checking whether the organoid matches its source and that there has not been contamination from other patients’ samples [35]. In addition, the importance of checking for mycoplasma contamination in media and organoid cultures is vital, as these prokaryotic organisms can affect the physiology of a cell and, thereby, potentially affect the experimental results [35,46,51].

#### 2.1.2. Setting up PDO Drug Screening Assays 

To utilize organoids in drug screening assays, cultured PDOs are harvested, organoid growth media is added to the cell suspension, and the cells are counted. The suspension is centrifuged and the cell pellet is then re-suspended in the gel-based matrix. The cells are seeded in a tissue culture plate and allowed to recover for two to three days (Figure 1A) [47,49]. After recovery, PDOs are exposed to media with different concentrations of drugs and incubated for six days. The effect of a drug is determined by measuring the cell viability after the drug treatment has ended [47,49]. Subsequently, the drug response observed with the organoid cultures is correlated with the clinical response observed in patients. This is achieved by correlating the area under the curve, maximal inhibitory concentration, and growth rate inhibition of PDOs to patient clinical response [52]. Clinical response measurements can be recurrence-free survival or progression-free survival [49]. 

Interestingly, the effect of radiation can also be evaluated using PDOs where the PDOs are exposed to radiation delivered by an irradiator. Irradiated PDOs are then allowed to recover for eight days to enable cell counting for cell viability measurements. PDOs that are not exposed to radiation are used as negative controls. The different treatment responses are then compared with clinical responses measured on tumor or patient level [49].

### 2.2. Establishment of Co-Culture PDOs

Owing to the fact that TME may affect drug response and that the stromal and immune cells found in the TME may affect the progression and immune evasion of the tumor, it is necessary to investigate treatment responses of PDOs comprising the various cell types found in the TME [14,15,53]. However, little research has been conducted in regard to co-culture methods. Notably, to generate PDO co-cultured with TILs, these cells should be expanded in vitro prior to the co-culture setup. It is possible to extract and expand TILs from the tissue specimen by fragmentation of the tissue (Figure 1). 

For the expansion of TILs, the tissue fragments are incubated in a growth medium supplemented with glutamine I, penicillin, streptomycin, HEPES, β-mercaptoethanol, human serum, and IL-2 [54]. Within one to two weeks after seeding, TILs may be observed as a dense cell layer around parts of the seeded tissue fragment, which can be cultured and maintained. In addition, it is possible to isolate peripheral blood lymphocytes (PBL) from whole blood (Figure 1B). The number of PDOs is counted to determine the number of TILs that need to be co-cultured. TILs are harvested, counted, and cultured in a ratio depending on the experimental design [54,55].

#### Setting up Co-Culture PDO Drug Screening Assays

It is vital to perform quality control before proceeding with co-culturing methods. By co-culturing PDOs with TILs, one can determine PDO killing by TILs following exposure to different treatments (Figure 1B) [36]. The tumor killing by TILs can be measured using a green fluorescent caspase probe, which binds to the DNA when the DNA is cleaved by caspase. Caspase activity is observed upon live imaging for three days and is used as a measure of cytotoxicity against PDOs. 

Alternatively, organoids and TILs are co-cultured for two weeks, and the specific subgroup of lymphocytes representing the CD8^+^ cytotoxic T cells is counted using a flow cytometer and/or the IFN-γ produced by these cells is measured to determine TILs’ activation [35,36,55,56]. Measuring cell viability of the co-culture model is not an option, as it is not possible to distinguish between viable PDOs or T cells.

## 3. Reviewing Organoid-Based Drug Screening Assays

The following section reviews the results of studies that have investigated the use of PDOs or co-culture PDOs as treatment selection models for pCRC and mCRC. 

### 3.1. PDOs

A study utilized 80 PDOs obtained from patients with rectal cancer (RC) to correlate the in-vitro treatment response with the clinical response when treated with radiochemotherapy with 5-FU. Remarkably, 68 out of 80 PDOs showed the same treatment response as the patient’s clinical response (Table 1) [48]. Another study involving patients with RC also investigated the correlation of clinical response with the PDOs response when treated with radiation only. The response observed in all seven PDOs was similar to the clinical response observed in patients, which was either no response, minimal, or complete response [49]. Both studies used the CellTiter-Glo 3D assay to measure cell viability by lysing the cells and measuring adenosine tri-phosphate (ATP). ATP is the energy currency of the cells and, by measuring ATP levels, one can estimate whether cells are dead or alive. One of the studies correlated PDO response at day 24 of exposure while the other correlated the same after six days (Table 1). Even though there are some differences in the assay design, these studies indicate that the use of PDOs for treatment selection and/or prediction when treating patients with radiotherapy is both a feasible and robust model.

Seven PDOs obtained from primary rectal cancer (RC) were treated with 5-FU and FOLFOX (5-FU and oxaliplatin). Notably, a 100% correlation of treatment sensitivity of PDOs to 5-FU and FOLFOX with progression-free survival in the corresponding patients was demonstrated [49]. In contrast, a study investigated the possibility of using PDOs to predict the response to FOLFOX in 10 patients and no correlation was found between PDO treatment response and clinical response for both combination and individual treatments [57]. Another study investigated correlation between in-vitro responses observed in PDOs from nine patients with mCRC treated with FOLFOX and clinical response in patients. They observed no significant difference in the drug response observed in the PDOs in patients with partial response or progressive disease [58]. These studies show conflicting results, where two out of three show no correlation between PDOs and patients’ clinical response when treated with FOLFOX. 

Ooft et al. explored the correlation of PDO drug response with clinical response in 10 patients with mCRC who were treated with irinotecan [57]. Irinotecan, a topoisomerase inhibitor, induces single-stranded breaks in the DNA and is frequently used in CRC treatment [4,60]. Five PDOs were obtained from tumors with progression and five from stable tumors. It was observed that PDOs from stable disease responded, while those from progressive disease did not. Furthermore, they investigated the response of 12 PDOs from patients treated with a combination of 5-FU and irinotecan. The response of all 12 PDOs corresponded to the clinical response observed in the patients [57]. 

Vlachogiannis et al. [47] correlated the TAS-102 response of three PDOs that originated from mCRC with patients’ clinical response. TAS-102 is composed of trifluridine, which inhibits DNA synthesis by incorporating into DNA strands and tipiracil, which promotes the inhibitory function of trifluridine [61]. TAS-102 has been approved as a treatment of mCRC when conventional treatment is ineffective. The response of all three PDOs correlated with the patients’ clinical response [47]. Vlachogiannis et al. also generated PDOs from a patient with one stable liver metastasis and two PDOs from progressive liver metastases. They observed that the response of all three PDOs matched the clinical response [47]. Therefore, this study indicates that PDOs have the potential to predict intra-patient heterogeneity. 

Further, Vlachogiannis et al. also compared the response of five PDOs established from mCRC with the patients’ clinical response when treated with cetuximab [47]. Cetuximab is a monoclonal antibody against the epidermal growth factor receptor (EGFR) that leads to cell growth arrest and suppression of cell survival [4]. The drug response of three out of five PDOs matched the clinical response [47]. 

It is important to note that all studies treated the PDOs for six to eight days, except for one study investigating radiation, where measurements were taken every third day for twenty-four days (Table 1). Cell viability was an endpoint measurement for all studies to evaluate the drug response of PDOs. Additionally, Yao et al. also measured the organoid size. All the studies were limited by the small sample size, between 4 and 19 patients, except for one study that examined 80 patients. However, the studies used slightly different methods to perform the PDO drug screening. In summary, this warrants the need to perform studies with larger cohorts and find a gold standard of the methodology to validate the findings.

More and more gel-based extracellular matrix products are emerging in the market, e.g., Matrigel and Basement Membrane Extract (BME), among others [52]. The stiffness of the gel-based extracellular matrix affects the formation of organoids by inhibiting the migration and motility of the cells [62]. In addition, the amount of Matrigel used during drug screening can also affect the efficacy of the drug, as a higher percentage of Matrigel can inhibit the localization of drug to the organoids [52]. This can potentially affect the results. The studies presented in this review use different concentrations of drugs, which could explain why the results are not consistent. They also do not assess whether the concentrations used correspond to what is obtained in vivo.

### 3.2. Co-Culture PDOs

Ramsay [36] investigated organoid killing by TILs extracted from pretreatment tumor biopsies. Twelve PDOs were established from patients with local disease and 20 PDOs from patients with mCRC. Organoid killing by TILs, measured by caspase activity, was significantly higher in PDOs from patients with a clinically complete response than that in patients with no response to therapy. Organoid killing by TILs was significantly higher in PDOs generated from mCRC than in those obtained from pCRC. Unfortunately, the study conducted by Ramsay et al. is only available as a meeting abstract, and therefore data regarding experimental design is limited [36].

In another study, 17 patients with metastatic RC received neoadjuvant chemoradiotherapy composed of 5-FU and radiation followed by surgery. Co-culture PDOs with TILs extracted from naïve tumor tissue specimens were successfully generated. Researchers investigated the ability of patients’ TILs to kill PDOs, thereby identifying those patients that most likely would respond to the therapy given to the patients. Co-culture PDOs from six patients who showed complete responses were shown to have a significantly higher organoid mediated killing than that from the patients who did not experience complete responses (Table 1) [59]. As previously mentioned, immune checkpoint inhibitors are a promising treatment. One study has investigated the activity of TILs obtained from PBL (based on IFN-γ levels) against organoids established from CRC tumors treated with nivolumab and ipilimumab. Co-culture PDOs were generated from six non-responders and six responders, where nine out of twelve were generated from naïve tumors. No TIL activity was observed against PDOs for any non-responders, and only three out of six co-culture PDOs generated from responders showed TILs activity [55].

So far, only three studies have investigated the efficacy of TILs using PDOs co-cultured with immune cells. The experimental design varied between these studies, where one study only measured the TIL-mediated killing while the other only measured the T-cell activity (Table 1). An important factor to take into consideration is whether the studies were generating organoids and extracting TILs from naïve specimens or already treated tissues. These three studies were based on small sample sizes, and therefore a larger cohort is needed to validate the finding that responders have a higher T-cell killing and T-cell activity.

## 4. Limitations

Despite the merits of this innovative in vitro technique, working with organoids reveals some limitations in that it is more expensive and time-consuming than monolayer cultures. The cell suspension obtained from the tissue specimen contains both healthy and cancer cells that are cultured together in an extracellular matrix. Healthy colon organoids usually have a higher growth rate than colon cancer organoids, which can result in the overgrowth of healthy cells influencing results during drug response examinations [63]. It is therefore essential to use selective media that is deprived of WNT when expanding the CRC organoids, as it will result in the growth of tumor organoids and thereby overcome the overgrowth of healthy cells [39]. 

As mentioned earlier, the mutational profile and protein expression of PDOs should be continually evaluated to ensure compliance. The histological similarity between PDOs and the original tumor also needs to be confirmed with IHC. These two methods support each other, as the characteristics of the genome and the interplay between cells is important to verify the presence of cancer. However, as the stromal compartment is not present in the organoid cultures, it can be difficult to assess with certainty whether the organoid originates from the cancer tissue or a closely related adenoma component. Evaluation of the cytological characteristics (variations in nuclei size and shapes, increased nuclei/cytoplasm ratio, hyperchromasia of the nuclei, and an increased number of mitoses) should be assessed; nonetheless, this cannot always compensate for the lack of stroma [64]. Notably, this issue has not been addressed in any study before and warrants more attention. A way to reduce this uncertainty could be to use a tumor from the surgical specimen for establishing the PDOs. This allows for obtaining a much larger amount of tissue from the central part of the tumor and ensures a higher chance of including the invasive cancer cells. However, this method is restricted by the fact that the tumor must be operable and is impossible in cases such as those with metastases, where surgery is not recommended.

Evidently, not all tumors can be generated into organoids, and tumors that are microsatellite instable, BRAF-mutated, or mucinous-like impose challenges [65]. Nevertheless, an improvement in the methodology of organoid culturing would help to overcome this issue and to achieve a higher success rate of PDO establishment. 

Organoids lack the TME, which influences treatment response, and therefore studies investigating the association of drug response in PDO co-cultures with patients’ clinical responses are necessary. New methods are being developed to co-culture PDOs with several other cell types found in the TME. A study has shown the possibilities of co-culturing fibroblasts expanded from primary tissue specimens of a pancreatic tumor with organoids and immune cells. Immunofluorescence analyses of organoids co-cultured with fibroblasts targeting smooth muscular actin (myofibroblast marker) and vimentin (fibroblast marker) revealed that both proteins were present [66,67]. This enables the maintenance of important cell types of the TME in PDO co-cultures and is a step further in our vision to closely mimic the real environment of the tumor. However, using this method with CRC organoids should be performed and verified before drug screening assays. It is necessary to explore whether this model is a superior predictor of clinical treatment response over PDOs and co-culture PDOs with TILs. Furthermore, to ensure the presence of the different TME cell types, additional validation of the co-culture organoids matching the corresponding tumor is necessitated. 

## 5. Potential

One of the main advantages of using PDOs in clinical and translational research is the recapitulation of the mutational profile and morphology of the primary tissue in organoids. Even after several passages of organoids, most of their characteristics are preserved [46]. Some researchers have observed changes in the genome during culturing; however, the CRC driver mutations were maintained between the primary tumor and corresponding organoids, and therefore the organoids continued to mimic the original tumor [37].

By linking the genomic data of a tumor to drug sensitivity data obtained from both PDOs and clinical evaluation, it is possible to obtain in-depth information regarding the cancer genotype and phenotype. This allows the assessment of a pharmacogenomic association to drug response and helps us fully understand the mechanisms in play, distinguishing the responders from the non-responders. 

Testing of new therapeutic drugs in clinical trials has a high failure rate, which can be eluded by using healthy PDOs as preclinical screening models [16]. Importantly, screening drugs using healthy PDOs may assist in identifying and evaluating the toxicity of novel drugs based on their detrimental or lethal effect on the healthy cells [68]. Additionally, by using PDOs as a treatment selection model, it may be possible to investigate the effect of combinational therapies. 

Potentially, PDOs should be generated from multiple biopsies from different areas of the tumor to investigate the response in a heterogeneous tumor [58]. This enables a more precise and patient-specific evaluation of the tumor [16]. This was incorporated by Narasimhan et al., where biopsies were pooled together, giving the advantage of obtaining a broader characterization, albeit with the limitation of granularity with respect to the spatial heterogeneity of the tumor [58].

PDO co-culturing enables the evaluation of the patient’s anti-tumor response of TILs against the tumor organoid. One study examined T-cell activity by measuring IFN-γ produced by TILs when co-cultured with PDOs; however, if the scope is to evaluate the ability of patients’ immune cells to kill PDOs, anti-tumor response should be the endpoint measurement (based on caspase activity). Therefore, more studies exploring the drug response of co-culture PDOs would potentially be of immense value. 

Two studies observed drug response of organoids established from pCRC and mCRC tumors obtained from the same patient [36,47], which highlights that generating PDOs from all tumors in a patient would assist in exploring intra-patient heterogeneity. Furthermore, it may help to consider whether different treatments are required to target the primary tumor and metastases or, even more so, whether differences between several metastases in the same patient are to be expected. 

A gold standard in performing drug-screening assays must be prepared to make the results across studies comparable. This includes, for example, whether organoids obtained from different patients are exposed to drugs at the same number of passages. This missing information can explain the varying drug response observed or the varying drug concentration used. In addition, the studies presented in this review use one endpoint measurement of PDO drug response, mostly ATP (Table 1). It could be of value if more endpoint measurements could be performed to confirm the reliability of the results. Collectively, these methodological limitations are easy to overcome by implementing a standardized procedure.

**Figure 3:**
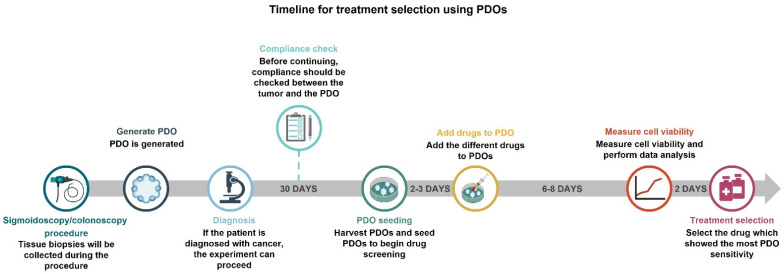
Estimated timeline for treatment selection using PDOs. Biopsies will be obtained at endoscopic procedures and a diagnosis will be made. Subsequently, PDOs will be generated from the biopsies. It is necessary to ensure compliance between PDOs and corresponding tissue before proceeding. After 30 days of culture, the harvested PDOs will be seeded for drug screening. After recovering for 2–3 days, they will be exposed to the library of drugs. Six days later, the cell viability will be measured, and data will be analyzed to select the appropriate treatment. PDOs, patient-derived organoids.

Ooft et al. [57]. and Ramsay [36] performed drug screening of PDOs and co-culture PDOs, respectively, within two weeks from PDO establishment, whereas Kong [59] reported a three to four week-long process of organoid establishment from biopsy sampling. The ideal process and timeline from diagnosis to treatment selection would be within three to four weeks; however, the methods need to be optimized to enable that. In Figure 3, the timeline of drug screening is outlined using the method described in this paper. To ensure a sufficient number of organoids, and if multiple drugs are investigated, the experiment would potentially take six weeks. 

In order to accomplish drug screening in a clinical timeframe, and due to the limited cell number after one week of PDO culture, it is necessary to determine one concentration of each drug to be tested that shows the highest chemosensitivity against organoids prior to its implementation in the clinic [57]. Technical replicates of PDOs exposed to drugs have shown low viability, which again suggests that it is a promising method [44,58]. To identify a personalized treatment strategy, several drugs must be evaluated, and this method allows for high-throughput drug screening which enables the simultaneous investigation of several drugs and, thereby, the possibility to stratify treatment options for individual patients. To avoid a delay in treatment from the day of diagnosis, the oncologists might initiate conventional therapy until the results of the drug screening are available. 

It is important to use this method in proximity to patients, which will allow the evaluation of PDO treatment in parallel with patients’ clinical care. Testing the effect of various drugs against PDOs before the start of patient treatment allows for prediction of the response of the corresponding tumor to individual treatment. If drug resistance is detected in the PDOs and/or clinical response, it could enable a quick change in treatment strategy and help avoid the unnecessary continuation of ineffective treatments with extensive adverse effects.

## 6. Conclusions

Despite the limitations of PDOs and co-culture with TILs, PDOs can be generated with high efficacy and show high compliance with the corresponding tumor. Twelve assays from five studies have examined the correlation of drug response of PDOs with patients’ response to treatment. Two studies showed no correlation of the response of PDO with clinical response when treated with FOLFOX. However, a tendency of the individual drug response of PDO to match the patient response was observed. Three studies examined the correspondence of PDO cytotoxicity and patient response when PDOs were co-cultured with TILs. The PDO co-culture method needs to be investigated further to predict clinical drug responses. The studies presented in this review indicate that PDOs obtained from pCRC and mCRC may be useful prediction models of patient response to therapy, even intra-patient heterogeneity. A standardized PDO culture method must be implemented to reduce procedural variability, making it possible for implementation in the clinic. Further, studies including larger patient cohorts are warranted in order to determine the suitability of PDOs in the clinic. Lastly, clinical trials using PDOs as a treatment selection method must be undertaken to conclude whether PDOs can be implemented in the clinic. 

## Figures and Tables

**Table 1:** Overview of studies correlating drug response of PDOs or co-culture PDOs with clinical outcomes in patients. pt, patient; pRC, primary rectal cancer; mCRC, metastatic colorectal cancer; mRC, metastatic rectal cancer; pCRC, primary colorectal cancer; PDO, patient-derived organoid; FOLFOX, 5-fluorouracil, and oxaliplatin; 5-FU, 5-fluorouracil; NA, not available; IHC, immunohistochemistry; CNV, copy number variation; NGS, next-generation sequencing; SNP, single nucleotide polymorphism; STR, short tandem repeat; ScEM, scanning electron microscope.

Reference	Cancer Type	Method	No of Organoids Investigated	No of pt Generated PDOs from	Investigating Intra-Pt Heterogeneity (Number of pt)	Treatment	Quality Control Check	Activation Before Assay	Time of Drug Testing	Endpoint	Endpoint Target	Endpoint Detection METHOD	No of PDO Correlating with Clinical pt Response	% Correlation Observed
Ganesh et al. [49]	pRC	PDO	7	7	0	5-FU	Exon sequencing, IHC	NA	6 days	Cell viability	ATP	CellTiter-Glo 3D	7	100
Ganesh et al. [49]	pRC	PDO	7	7	0	FOLFOX	Exon sequencing, IHC	NA	6 days	Cell viability	ATP	CellTiter-Glo 3D	7	100
Ganesh et al. [49]	pRC	PDO	19	7	NA	Radiation	Exon sequencing, IHC	NA	8 days	Cell viability	ATP	CellTiter-Glo 3D	7	100
Yao et al. [48]	pRC	PDO	80	80	0	5-FU and radiation	IHC, CNV	NA	24 days	Organoid size, cell viability	Size (uM), ATP	Image-Pro Plus 6.0, CellTiter-Glo 3D	68	85
Vlachogiannis et al. [47]	mCRC	PDO	6	4	1	TAS-102	IHC, NGS	NA	6–8 days	Cell viability	Metabolic capacity	CellTiter-Blue	4	100
Vlachogiannis et al. [47]	mCRC	PDO	5	5	0	Cetuximab	IHC, NGS	NA	6–8 days	Cell viability	Metabolic capacity	CellTiter-Blue	3	60
Ooft et al. [57]	mCRC	PDO	10	10	0	Irinotecan	SNP	NA	6 days	Cell viability	ATP	CellTiter-Glo 3D	10	100
Ooft et al. [57]	mCRC	PDO	12	12	0	5-FU and irinotecan	SNP	NA	6 days	Cell viability	ATP	CellTiter-Glo 3D	12	100
Ooft et al. [57]	mCRC	PDO	16	10	0	FOLFOX	SNP	NA	6 days	Cell viability	ATP	CellTiter-Glo 3D	0	0
Ooft et al. [57]	mCRC	PDO	16	10	0	5-FU	SNP	NA	6 days	Cell viability	ATP	CellTiter-Glo 3D	0	0
Ooft et al. [57]	mCRC	PDO	16	10	0	Oxaliplatin	SNP	NA	6 days	Cell viability	ATP	CellTiter-Glo 3D	0	0
Narasimhan et al. [58]	mCRC	PDO		9	3	FOLFOX, FOLFIRI	STR, IHC	NA	6 days	Cell viability	ATP	CellTiter-Glo 2.0	0	0
Kong et al. [59]	mRC	Co-culture PDO	17	17	0	5-FU and radiation	STR, IHC	NA	3 days	Killing assay	Caspase 3/7, Propidium Iodide	Caspase activity, ScEM	17	100
Chalabi et al. [55]	pCRC	Co-culture PDO	13	12	1	Nivolumab and ipilimumab	SNP	Organoid with IFN-g	14 days	T-cell activity	IFN-γ	Cytometric Bead Array	9	75
Ramsay [36]	pCRC	Co-culture PDO	12	12	NA	NA	NA	NA	NA	Killing assay, T-cell activity	Caspase, IFN-γ	Caspase activity, NA	NA	NA
Ramsay [36]	mCRC	Co-culture PDO	20	20	NA	NA	NA	NA	NA	Killing assay, T-cell activity	Caspase, IFN-γ	Caspase activity, NA	NA	NA

## Data Availability

Upon reasonable request to the corresponding author, data are available.

## Supplementary Materials

The following are available online at https://www.mdpi.com/article/10.3390/cancers14041069/s1, Table S1: Reagents and solutions needed for organoid medium.
