# Supplementary material for: Use of Patient-Derived Organoids as a Treatment Selection Model for Colorectal Cancer: A Narrative Review
Source: Cancers (Basel). 2022 Feb 20;14(4):1069. doi: 10.3390/cancers14041069 (PMC8870458; doi:10.3390/cancers14041069)
Supplement: Supplementary file 1 [file cancers-14-01069-s001.zip › cancers-1584779-supplementary.pdf]

Use of Patient-Derived Organoids as a Treatment Selection Model for Colorectal Cancer: A Narrative Review

Sara Furbo, Paulo César Martins Urbano, Hans Henrik Raskov, Jesper Thorvald Troelsen, Anne-Marie Kanstrup Fiehn and Ismail Gögenur

Table S1. Reagents and solutions needed for organoid medium.

**Reagents and solutions**
DMEM/F12
Ultraglutamine I
HEPES buffer
Pensicillin
Streptomycin
Noggin
R-Spondin1
B27 without vitamin A
N-acetulcysteine
Nicotinamide
Human EGF
ALK5 inhibitor (TGF-β Type I Receptor inhibitor)
P38 inhibitor
Prostaglandin E2
WNT
